# Popular Gym Fitness Sport: An Analysis of 1387 Recreational Athletes Regarding Prone to Pain Exercises and the Corresponding Localisations

**DOI:** 10.3390/sports12010012

**Published:** 2023-12-29

**Authors:** Maria A. Bernstorff, Norman Schumann, Andreas Finke, Thomas A. Schildhauer, Matthias Königshausen

**Affiliations:** 1 Department of General and Trauma Surgery, Arthroscopic and Sports Orthopaedic Section, Bürkle—de—la Camp 1, 44789 Bochum, Germany; a.finke@gmail.com (A.F.); thomas.schildhauer@bergmannsheil.de (T.A.S.); matthias.koenigshausen@gmail.com (M.K.); 2 Institution for Mathematics, Ruhr University, 44789 Bochum, Germany; norman.schumann@rub.de

**Keywords:** fitness, sports injuries, prevention, weightlifting, muscle training

## Abstract

Background: Recreational fitness sports are popular worldwide and rank first among organised sports. This study aims to bridge a knowledge gap by examining which exercises are most prone to causing pain symptoms, as a possible precursor for injury, and analysing the body regions that are most frequently affected. Methods: Using an online questionnaire, 20 demographic and training-specific items and 49 sport-specific exercises were recorded. Frequent exercises as well as the incidence and distribution of pain symptoms that the athletes experienced during or in relation to their training were evaluated. Results: The study assessed common exercises and documented the frequency and distribution of pain symptoms experienced by athletes during or in relation to their training. A total of 1387 respondents were included in this study. Of these, 732 (53.1%) experienced pain during their fitness training, with 333 (24.2%) being female and 397 (22.3%) being male. The method of creating a training plan showed a significant influence (*p* < 0.001): athletes who devised their own plans reported pain or instability more frequently than those in the comparison groups. Guided exercises on machines resulted in the lowest frequency of pain (11.54%), while exercises with free weights were associated with the highest pain rate among respondents (19.94%). Specifically, exercises such as the back squat, deadlift, bench press, and triceps dips were identified as the exercises most commonly associated with pain. The most frequently reported pain region was the shoulder, followed by the lower back and knees. Conclusion: The findings reveal a significant number of unreported pain symptoms. The disparity between rigorous training volumes and the absence of professional care frequently leads to injuries and pain. It is incumbent upon sports medicine to investigate the root causes of these complaints (pain or instability) to implement preventive measures against potential injuries.

## 1. Introduction

In recent years, the number of ambitious amateur athletes in fitness and weight training has continued to increase, sometimes training at a level akin to that of professional athletes. According to a survey conducted by a German health insurance company, 36% of the population reported their involvement in gym training gym [1]. Many gyms, especially those in the lower price range, offer 24/7 workout options. A study by “Deloitte” revealed that the coronavirus pandemic and the subsequent closure of fitness studios resulted in a decrease in the annual growth rate of gym memberships. Nevertheless, fitness sports still hold the top spot among association- and club-organised sports [2]. The largest age group of gym users falls between 20 and 29 years old, but even in the 70-plus age group, approximately 1.3 million people actively participate in fitness facilities in Germany as an example [1].

However, in many cases, training is focused solely on achieving a “good look” without a proper understanding of how it may harm the body. This can lead to the development of stress syndromes, which often require a complete cessation of training or the elimination of specific exercises to manage them. Stress syndromes may even result in chronic pain, fatigue fractures, and other musculoskeletal injuries [3,4].

In research, a limited number of studies with a similar design that specifically focused on recreational fitness sports were encountered. However, some studies delve into this topic within specific population groups, such as sports students, or concentrate on particular exercises [3,5,6,7,8,9,10,11]. Various authors concur that athletes frequently exhibit distinct injury patterns, necessitating customised therapeutic approaches. Consequently, terms like “bench-presser’s-shoulder” have become well-established in the literature [7,9,12]. It is not uncommon to encounter individuals who, after years of training specific muscle groups with particular exercises, can no longer perform them due to pain or injury [5,7]. Complaints in the form of pain, restricted range of motion, or instability often serve as initial symptoms preceding the development of manifest injuries and should be treated seriously as warning signals.

Due to the absence of large-scale studies involving recreational athletes who lack proper trainer supervision or a fundamental understanding of exercise science, it is reasonable to assume that a significant number of injuries and overuse syndromes go unreported. The primary objective of this study is to address this knowledge gap. The study focused on identifying exercises that are most likely to lead to pain among these athletes and examines the distribution of pain localisations. Another objective is to identify approaches for enhancing training methods and establishing a scientific foundation for developing therapy concepts to effectively address sport-specific injuries and pain. Based on current knowledge, a comprehensive analysis of these specific exercises has not yet been undertaken in current scientific research. Thus, the hypothesis of this study is that a significant number of recreational athletes suffer pain more frequently than expected. It is assumed that the causes of the pain accumulate during individual exercises.

## 2. Materials and Methods

A questionnaire was invented using the online tool “www.umfrageonline.com (accessed on 4 December 2023)”. This questionnaire comprised 20 demographic items and 49 exercises specific to training. The selection of exercises was made in collaboration with resident gym trainers to ensure a representative cross-section of the exercises most commonly performed in gyms.

The survey was conducted online and anonymously. All respondents were presented with the privacy policy and voluntarily agreed to participate in the study. Individuals of all genders and ages had the opportunity to take part in this survey. Informed consent was obtained from all participants or their legal guardians as necessary. The questionnaire was disseminated through various channels, including social media platforms. Prominent gym chains, such as FitX^®^ (a leading gym chain in Germany), shared the survey link with their members, newspaper articles were published to raise awareness, and multiple physical therapy and medical associations actively participated in distributing the survey. Furthermore, support for this study was provided by organisations such as the Functional Fitness Association, CrossFit^®^ Boxes, and the Bodybuilding Association Germany^®^.

Before conducting the study, approval was obtained from the local ethics board (Ethics Committee number: 18-6729 BR).

### 2.1. Questionnaire

To encompass a diverse range of individuals within the gym community, no specific age limit was established. The objective was to include all recreational athletes. Accordingly, results were classified and distinguished during analysis, taking into account athletic training methods, training intensities, and levels of athletic experience.

Initially, demographic information pertaining to each individual was gathered. Subsequently, training-related parameters were assessed (refer to Table S1 in the Supplementary Materials). This included recording the age, gender, height, and weight of each participant. Training-specific data encompassed the number of years spent in training, weekly training frequency, total weekly training hours, the method of devising the training plan (self-made, online, by a trainer, or none), regular engagement in other sports, and if so, whether these sports were pursued at a competitive level. Additionally, the performance of a regular warm-up routine was noted.

Furthermore, participants were queried about their smoking habits and the physical demands of their work, categorised as physically highly active (>8 h), physically active (4–8 h), physically moderately active (2–4 h), or physically inactive (0–2 h). In the exercise-specific section, respondents were initially asked whether they had ever experienced pain or movement limitations related to their gym training. If the response was negative, they were directed straight to the final set of questions. If the initial query was answered affirmatively, specific exercises were presented for assessment.

For each exercise, participants had the opportunity to indicate the following:

(1) Whether they performed the exercise and experienced pain but continued despite the pain. If they did not stop the exercise immediately but suffered pain, they were asked whether they stopped it after a defined period (e.g., after 4 weeks, less than one year, more than one year).
(2) Whether they performed the exercise without any health issues.
(3) Whether they never performed the exercise.

**Figure 1:**
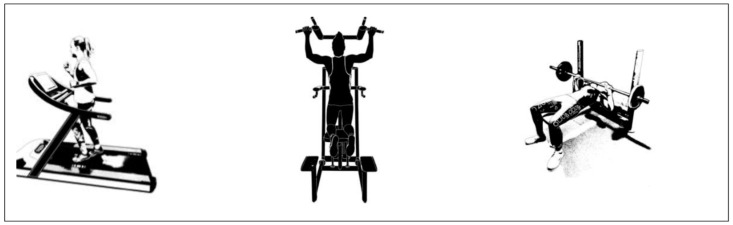
Examples of pictograms (from left to right: cardio: treadmill; guided machine exercise: assisted pull-up; free weight: bench press).

To prevent any confusion regarding exercise names and techniques, all exercises were depicted using pictograms developed exclusively for this study, illustrating how each exercise was performed (Figure 1).

**Figure 2:**
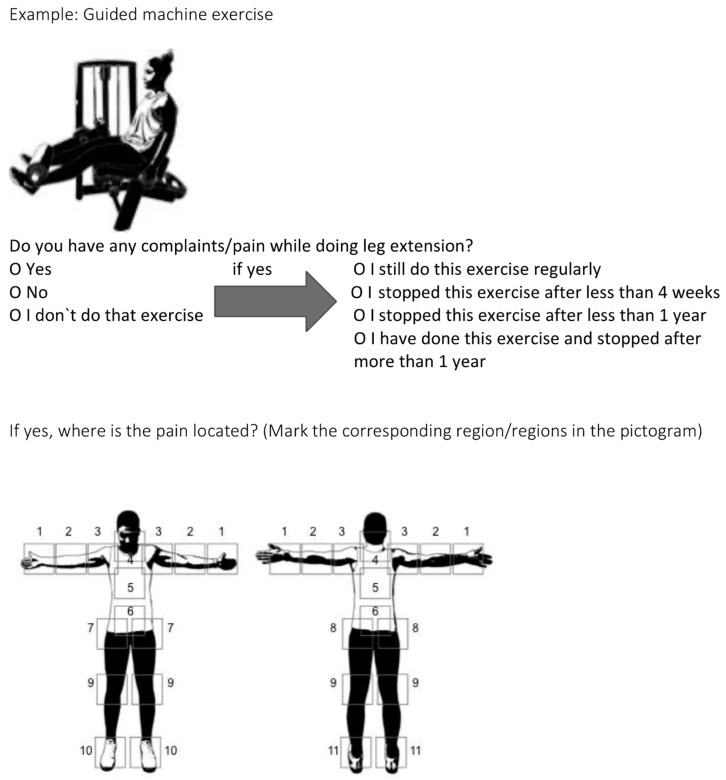
Example of question for all 49 exercises (guided machine exercise: leg extension; pain localisations: (1) hand/wrist, (2) elbow, (3) shoulder, (4) neck/cervical spine, (5) thoracic spine, (6) lower back/lumbar spine, (7) hip, (8) sacroiliac joint, (9) knee, (10) foot, (11) ankle.

All 49 exercises were classified into four categories: bodyweight, free-weight, guided machine exercises, and cardio machines. For each exercise, the initial inquiry focused on whether the exercise was part of the participant’s routine and, if so, whether any issues were encountered while performing it. Subsequently, to gauge the severity of any pain or discomfort, respondents were asked whether they persevered with the exercise or had to discontinue it after a certain duration (Figure 2).

In the event of an affirmative response, participants were queried about the specific location of the pain. Eleven predefined regions were identified and presented through a one-to-one correspondence in a pictogram format. This simplified the process for respondents, allowing them to select one or more affected regions and minimising potential misunderstandings regarding the response options (Figure 2).

### 2.2. Statistical Analysis

For the determination of sample size, a principle commonly employed in collecting representative populations for general surveys was utilised. According to current data, there are approximately 9.3 million athletes in German gyms. Employing a 95% confidence level and a 3% margin of error, the calculation necessitated a sample size of at least 1067 athletes, which significantly exceeded the size of the population assessed in this study. The margin of error was deliberately kept low at 3%. Throughout the study, the chi-square test (either Pearson or Fisher, depending on the sample size) was employed as the statistical method to assess stochastic independence in contingency tables. The statistical characteristics X and Y of the null hypothesis “H0: Characteristics X and Y are stochastically independent” are detailed in the text. The *p*-value was reported when the null hypothesis was significantly rejected at an alpha level of 0.05. Statistical analyses were performed using Python 3.8 (Python Software Foundation. Python Language Reference, version 2.8, 9450 SW Gemini Dr., ECM 90772, Beaverton, OR 97008, USA) and Jupyter 1.0.0 (Jupyter Notebooks, 2016, OR 97008, USA). The packages utilised for calculations and visualisations included pandas 1.2.0, numpy 1.19.4, seaborn 0.11.1, and scipy 1.5.4 (Python Software Foundation. Python Language Reference, version 2.8, 9450 SW Gemini Dr., ECM 90772, Beaverton, OR 97008, USA).

The datasets generated and/or analysed during the current study are available upon reasonable request from the corresponding author. The datasets generated during and/or analysed during the current study are available from the corresponding author upon reasonable request.

## 3. Results

A total of 1387 respondents participated in the study, comprising 719 females (52.2%), 663 males (47.4%), and five individuals (0.4%) of undisclosed gender (Table 1). The study also encompassed data on age, body mass index (BMI), and body fat.

Out of the respondents, 155 (11.2%) were smokers, while 1232 (89.4%) reported not smoking regularly. The largest group of respondents, numbering 506 (36.7%), had been engaged in gym workouts for 3 to 7 years. The most commonly reported workout frequency was 3 to 4 times per week. Total weekly workout hours were divided into four categories, with 620 individuals (45%) exercising for 5 to 10 h weekly. The range of total weekly workout hours varied from 1 to 35 h, with an average of 4 h.

In total, 925 athletes (67.1%) followed a training plan. Among them, 477 (51.6%) created their training plan independently, 406 (43.9%) had a trainer design their plan, and 42 (4.5%) followed an online training program (Table 1).

Overall, 732 respondents (53.1%), consisting of 333 females (45.5%) and 397 males (54.2%), reported experiencing or having experienced pain related to their gym workouts. A total of 655 respondents (47.5%) denied experiencing pain. Gender played a significant role in these findings (*p* < 0.001) (Table 2), with women reporting fewer pain symptoms related to their fitness training compared to men. No significant correlation was observed with age (*p* = 0.1892) (Table 2). 

Interestingly, individuals falling into the overweight and obese categories were significantly more likely to report pain compared to those with normal or underweight statuses (*p* = 0.046) (Table 2). Furthermore, there was a significant association between the duration of training in years (*p* < 0.001) (Table 2) and the likelihood of pain symptoms. Respondents with over 8 years of training experience exhibited a higher prevalence of pain symptoms (*n* = 289, 20.9%), followed by those with 3 to 7 years of training (*n* = 274, 19.9%), 2 to 3 years of training (*n* = 95, 6.9%), and 1 to 2 years of training (*n* = 74, 5.4%).

The frequency of training sessions per week also had a significant impact on the likelihood of experiencing pain in connection with fitness training. Particularly, those engaging in 4 to 6 training sessions per week showed a significantly higher incidence of pain (*p* < 0.001) (Table 2).

Interestingly, the manner in which the training plan was established also exhibited a significant influence (*p* < 0.001) (Table 2). Athletes who independently created their training plans reported pain or instability more frequently than those in the comparison groups. Furthermore, engaging in regular physical activity in addition to fitness training proved to be beneficial, as athletes who also participated in other sports reported fewer pain symptoms associated with fitness training (*p* = 0.026) (Table 2).

### 3.1. Exercise and Pain Association

Table 3 reveals that among amateur athletes, the most common exercises leading to pain include the back squat, deadlift, bench press, and triceps dips. It is worth noting that these exercises were not the most frequently performed ones, although they all fall under the category of free-weight exercises. In the cardio equipment category, running on the treadmill also stood out as a frequent source of problems relative to the number of individuals performing it.

When comparing exercises targeting the same muscle groups (e.g., bench press with a barbell and pectoral press on a machine), the level of discomfort was significantly higher with free-weight exercises, both in terms of the degree of pain and the overall number of incidents. As outlined in the methods section, each individual exercise is categorised (body weight, free-weight training, cardio, guided machine exercises). To analyse the relationship between exercise frequency and the number of athletes performing them, the percentage of each category contributing to these pain symptoms was calculated.

It is noteworthy that guided machine exercises were associated with the least frequent pain indications among athletes (11.54%), followed by training on cardio equipment (12.8%). Conversely, exercises involving free weights were the most likely to cause pain among the respondents (19.94%), closely trailed by bodyweight exercises (18.31%).

### 3.2. Pain Localisation

**Figure 3:**
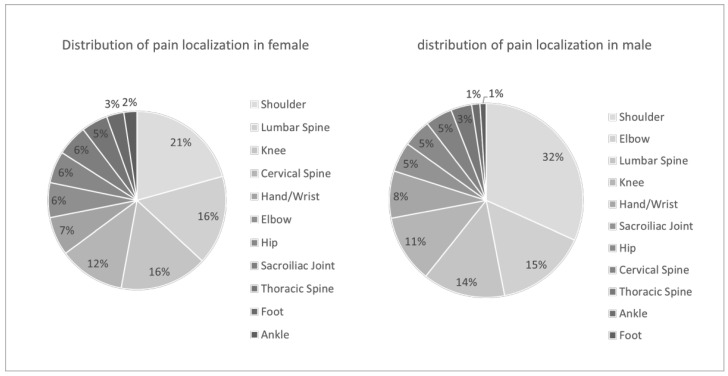
Distribution of pain localisation in female and male respondents, with a significantly higher percentage in male athletes.

To analyse the distribution of pain in the context of fitness training, a total of 11 specific locations were defined. Notably, the shoulder region was the most commonly affected, with 1114 cases (696 males, 62.5%, and 417 females, 37.4%), followed by the lumbar spine with 632 cases (303 males, 48%, and 328 females, 52%). The most significant difference between genders was observed in reports of pain at the elbow, with 463 cases (335 males, 72.4%, and 128 females, 27.6%). Additional values can be found in Figure 3.

As illustrated in Figure 3, there was a distinct variation in the distribution of pain locations between males and females. The shoulder joint remained the most prevalent site of pain in both genders, but the percentage is notably higher among male athletes. In general, there was a greater impact on the upper extremities and lower back among males, while females experience increased impact in the lower extremities, neck, and lower back (Figure 3).

### 3.3. Gender Differences in Training Behaviour

**Figure 4:**
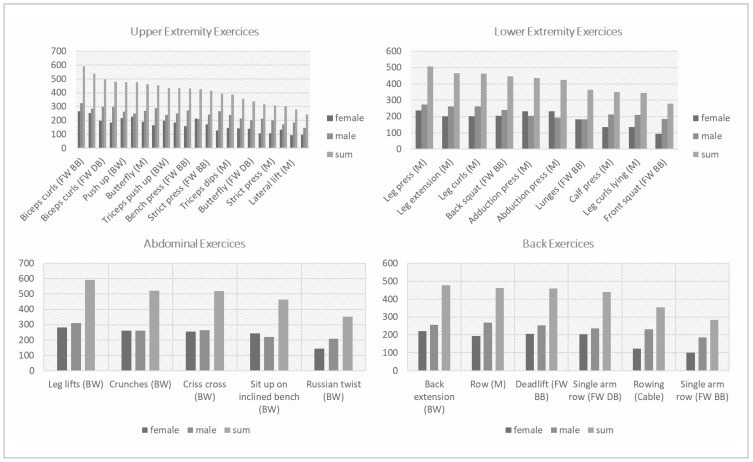
Consideration of gender differences in exercise behaviour in relation to upper body, lower body, abdominal, and back exercises.

To explore potential reasons for gender differences in pain localisation, various exercise categories were analysed. Figure 4 presents upper extremity exercises arranged in descending order and divided by gender. It is evident that there was a notable overrepresentation of upper extremity exercises among men, and men are more frequently involved in nearly all exercises compared to women in this category.

In contrast, when it comes to lower extremity exercises, the gender distribution was more balanced than in upper extremity exercises. Notably, guided exercises that were less prone to injury have attracted a larger number of participants (Figure 4). Exercises targeting the back also exhibited a higher proportion of male participants (Figure 4). Conversely, the gender ratio in abdominal exercises tended to be more evenly balanced (Figure 4).

Free-weight, weight, and guided machine exercises

**Figure 5:**
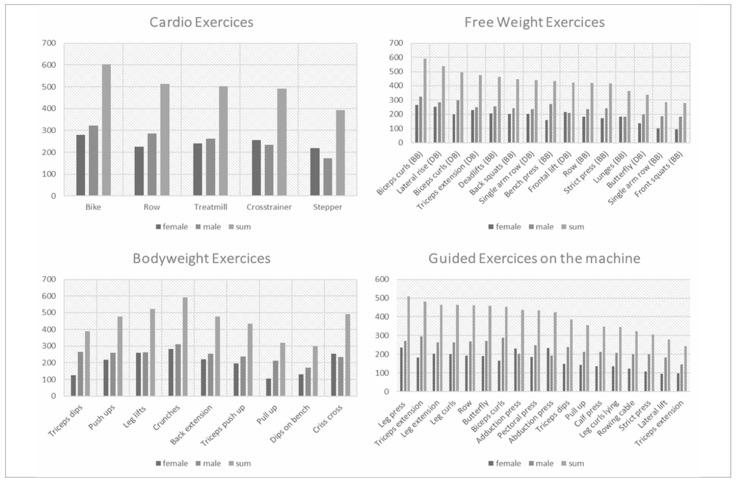
Consideration of gender differences in exercise behaviour in relation to cardio, free-weight, bodyweight, and guided exercises on the machine.

As previously detailed in the Results section, it becomes evident that free-weight exercises, closely followed by bodyweight exercises, exhibited the highest rates of injury. In the realm of cardio training, there were no significant gender differences among athletes, with only a few exceptions in specific exercises. Male athletes, as shown in Figure 5, clearly favour the use of free-weight exercises more frequently. This preference extended to bodyweight exercises, particularly those targeting the upper extremities. Notably, there was also an increased utilisation of machine training by male athletes (Figure 5).

At the conclusion of the survey, respondents were asked if they had forgotten to mention any exercises, to which only 54 individuals (2.12%) responded affirmatively. Notably, the exercises mentioned by these athletes were rarely identified by name. This underscores that the exercise selection provided by the authors constitutes a representative sample of exercises typically performed in the gym.

## 4. Discussion

Fitness sports as a leisure activity are among the leading sports worldwide and rank first in Germany, as an example. This study, conducted through a nationally distributed online survey, focused on identifying the most common gym exercises associated with pain and analysing the distribution of pain by body region. The hypothesis of this study was that a significant number of recreational athletes suffer pain more frequently than expected. It is assumed that the causes of the pain accumulate during individual exercises. In the field of sports medicine, it is well-established that chronic pain symptoms often result from repetitive microtrauma [3,4,13,14]. While certain sports are prone to acute injuries, particularly extreme sports with frequent accidents [6,15,16,17,18], others are characterised by the development of chronic pain or instability due to repetitive microtrauma. Although acute accidents can occur when using weights [6], they are typically the exception, with chronic pain or instability arising more frequently due to misuse and overuse.

Out of the 1378 respondents, 52.5% reported experiencing pain or instability related to their fitness training in a gym, while 47.4% denied such complaints. Pain and feelings of instability associated with fitness training can be harbingers or existing signs of injury. The very high level of pain reported here underlines the hypothesis that there are significantly more occult injuries or at least pain in popular fitness sports than previously assumed. In summary, gender, the way in which the training plan was designed, the practice of other sports, obesity, the training frequency per week, and training experience over the years can be identified as significant influencing factors. Furthermore, free weights and bodyweight exercises show higher pain rates in the recreational athletes concerned than guided exercises on the machine and the use of cardio equipment. The exercises most prone to pain are the back squats, deadlifts, bench press, and dips. The shoulder joint is significantly more frequently affected than other parts of the body, followed by the lower back and knee joint. 

A significant positive influence of gender was observed on the absence of pain (*p* < 0.001), women reporting fewer pain symptoms related to their fitness training compared to men), which aligns with findings from other studies examining gender differences in sports [5,7,11,19,20]. The design of a training plan appeared to play a critical role in pain prevention. Athletes following training plans created by coaches reported fewer instances of pain compared to those who independently designed their training plans. This is an indication that professional supervision alone leads to a reduction in possible injuries in fitness sports. Moreover, it is worth noting that free-weight and bodyweight exercises were more likely to lead to issues than cardio exercises or machine exercises (Figure 5) [14,21].

Athletes who independently created their training plans report pain symptoms significantly more frequently than the comparison groups (self-made plan vs. plan made by a trainer, *p* < 0.001). This intriguing finding underscores the importance of extending sports science and medical support to recreational athletes as well. The mere fact that a training plan is professionally prepared and effectively communicated could potentially reduce the incidence of pain among these athletes.

Engaging in regular sporting activities alongside fitness training had a notably positive impact (*p* = 0.026). Athletes who participated in other sports, in addition to their fitness training, tended to report fewer pain symptoms related to their fitness training. Other research groups see a possible explanation at the neurophysiological level [22]. However, it is important to note that this observation requires further prospective investigation for comprehensive substantiation. One could speculate that individuals who were involved in other sports during their youth and received proper coaching exhibit distinct training behaviours. Furthermore, it is conceivable that engaging in complementary sports, as is common among professional athletes, may contribute to injury reduction through varied forms of physical stress and loading [23].

It is noteworthy that guided machine exercises resulted in the lowest number of pain indications among athletes (11.54%). This was followed by training on cardio equipment (12.8%). Conversely, exercises involving free weights elicited the highest number of pain indications among respondents (19.94%), with exercises using body weight closely following (18.31%).

Given this context, athletes in the gym are often advised to prioritise guided exercises because they allow for minimal or no faulty execution. However, to the best of our knowledge, this statement has yet to be scientifically substantiated. Consequently, it remains inconclusive whether free-weight training, when performed with proper technique and appropriate weight, leads to more pain than guided exercises on machines (also executed with correct technique and similar weight). Nevertheless, for the general gym-going population, it can be posited that pain resulting from machine-based training tends to be lower. At this point, further investigations with regard to neuromuscular complexity would certainly be useful. Based on the data presented here, it could be assumed that a high CNS impact during the performance of an exercise can lead to pain or a feeling of instability in the athlete.

The notably high pain data associated with bodyweight exercises are cause for concern as well. From our perspective, these exercises are frequently underestimated. During bodyweight exercises, athletes must move their entire body weight, which is non-negotiable. For instance, in a free pull-up, an athlete lifts their own body weight (e.g., 80 kg) on a bar. A load that may not necessarily be matched when using a barbell in a strict press or during incremental weight increases. The findings of this study indicate that exercises such as back squats, deadlifts, bench presses with a barbell, and triceps dips are the most injury-prone exercises (Table 2). This implies that these particular exercises possess a kinematic complexity that necessitates diligent supervision by an experienced coach to optimise the range of motion, tempo, and appropriate weight intensity. Moreover, the progression from set to set should be tailored to the athlete’s abilities.

The primary objective of this study is to identify the body regions most commonly afflicted by pain in relation to fitness training. The data showed a significant difference in the frequency of pain and gender. It therefore seems sensible to analyse the results on a gender-specific basis as well. The findings of this study reveal that the shoulder is overwhelmingly the most frequently affected region, followed by the lumbar spine, the knee region, the elbow, the cervical spine, the hand/wrist, the sacroiliac joint, the hip, the thoracic spine, the foot, and finally, the ankle joint. These findings are similar to other sports in the field of weight training [5,10,12,24,25,26,27,28,29].

An analysis of the distribution of pain locations reveals discernible distinctions between genders. Although both sexes commonly experience shoulder joint pain, there is a noticeable shift in the distribution towards the upper extremities among males and towards the lower extremities among females. Additionally, spinal discomfort, particularly in the lumbar and cervical regions, is more prevalent among females, although lower back pain is also frequently reported by males [30].

As evident from the results section, notable differences exist in the design of training plans between both genders. Both in terms of targeted body regions and exercise categories, it becomes apparent that females engage in fewer classic strength exercises and more cardiovascular training and abdominal exercises (Figure 4 and Figure 5). This trend may align with the higher prevalence of lower extremity and lumbar/spine pain among females.

It is important to acknowledge a limitation of this study, namely, the inability to assess the intensity of individual exercises. This includes factors such as the amount of weight used in strength exercises, the number of sets, and the repetitions per set. These aspects would likely serve as additional influencing factors in the analysis of various injury regions. Furthermore, an intriguing question that could have been explored is the training intent. For instance, it could be postulated that women may prioritise training for health or achieving a slim physique, while men might lean towards building muscle mass rapidly, potentially involving the use of heavier weights, which could carry a (potentially) higher risk of injury.

Traditional fitness sports exercises are often incorporated into other athletic disciplines. Several studies have investigated the incidence of injuries in activities such as CrossFit^®^ or weightlifting [3,24,25,31,32]. Notably, the repertoire of free-weight and body weight exercises frequently overlaps with those encountered in weightlifting, Strongman^®^, CrossFit^®^, or High-Intensity Interval Training (HIIT). Research in this area similarly identifies the shoulder joint as the most commonly affected joint, with the lower back and knee closely following, albeit with some variation in the order depending on the literature [21,24,31,33,34]. It is worth noting that training in these sports typically follows a more club-like structure and is often guided by coaches.

### 4.1. Limitations

Regarding limitations, it is important to note that the data and analyses were obtained from a cross-sectional study. This makes it challenging to draw individual-level conclusions. Additionally, the recruitment process, conducted online on a voluntary basis, may have introduced selection bias. This bias could arise from excluding individuals without internet access and potentially attracting respondents with a particular interest in the topic.

Furthermore, it is worth acknowledging that not all possible exercises were included in the study, providing a somewhat limited view of the entire spectrum of fitness exercises. However, it is important to note that the selection of exercises was made in consultation with fitness trainers from frequently visited gyms and aligns with the preferences of the majority of gym goers. This is supported by the fact that only a small portion of respondents (*n* = 54, 3.9%) indicated that some exercises were missing out of a population of *n* = 1387, suggesting that the exercise selection was appropriate.

The specification of body regions in the study is intentionally general. This choice was made because respondents may not have been able to make more specific distinctions without professional or medical guidance. Therefore, we deliberately chose not to differentiate, for example, the shoulder into anterior/posterior shoulder or clavicle region or provide detailed anatomical structures (e.g., long head of biceps).

### 4.2. Clinical Relevance

It is important to highlight that, despite 53% of the surveyed athletes reporting pain or instability during fitness sports, a substantial 47% of participants stated that they had never encountered any issues during their fitness activities. It is worth noting that fitness sports are widely recognised for their positive impact on overall physical and mental health, representing a significant contributor to well-being.

The data from this study imply that engaging in fitness sports should be done with an understanding of potential problems and in moderation to prevent long-term damage, thereby allowing this sport to yield its positive effects over the athlete’s lifetime. Considering that, according to a survey, 9.26 million people in Germany are enrolled in gyms, it is plausible that over half of them have experienced or are currently dealing with pain. This underscores the urgent need for more extensive research in sports medicine, focusing on prevention, diagnosis, and therapy, in order to advance our understanding beyond current levels.

## 5. Conclusions

The present study marks the first extensive examination of injury patterns among recreational athletes participating in fitness sports. The results, collected through a nationwide online survey in Germany, shed light on a substantial number of unreported injuries within this sport. Notably, the study underscores the significance of a professionally crafted training plan, emphasising its potential to prevent initial pain and recurring symptoms in athletes. Male athletes report pain more frequently than their female counterparts. This should be followed by studies that analyse whether men train beyond their strength capacities and overexert themselves more often. Among exercises, back squats, deadlifts, barbell bench presses, and triceps dips emerge as the exercises with the highest risk of injury. In contrast, exercises performed on fitness machines tend to cause less pain compared to those involving free weights or body weight. This suggests that inexperienced athletes in particular should include machine-based exercises in their training plan.

The study reveals that the shoulder is the most commonly affected body region, followed by the lumbar spine, knee region, elbow, cervical spine, hand/wrist, sacroiliac joint, hip, thoracic spine, foot, and finally, the ankle joint. As other studies have also shown, the shoulder, as a muscle-guided joint, is most frequently affected by pain and injuries and should include special exercises to strengthen the rotator cuff, especially during upper body training. Professional training support is particularly important here. Notably, clear gender differences in the distribution of pain locations are evident. The data underscore a disconnect between the often-substantial training volume and the absence of professional guidance, which frequently results in injuries and pain among athletes. Addressing the causes of these pain symptoms should be a paramount objective of sports medicine to implement preventive measures against potential injuries. This study serves as a broad foundation for future in-depth investigations into individual exercises.

## Figures and Tables

**Table 1:** Personal and training-specific data collected in the questionnaire (translated into English for publication).

Personal Data		
**Sex Distribution**		
	Male	*n* = 663
	Female	*n* = 719
	Diverse	*n* = 5
**Age Groups**		
	Under 20 years	*n* = 11
	20–30 years	*n* = 482
	30–45 years	*n* = 484
	45–60 years	*n* = 251
	Over 60 years	*n* = 150
**BMI**		
	underweight: </=18.5	*n* = 28
	normal/ideal: 18.5–25	*n* = 733
	overweight: 25–30	*n* = 438
	obese: 30–35	*n* = 120
	extreme obese: >/=35	*n* = 58
**Body Fat Percentage (If Known)**		
	</=10	*n* = 58
	10–20%	*n* = 243
	20–30%	*n* = 109
	>30%	*n* = 41
**Training Specific Data**		
**Years of Training in a Gym**		
	1–2 years	*n* = 207
	2–3 years	*n* = 194
	3–7 years	*n* = 506
	>/=8 years	*n* = 480
**Frequency of Training per Week**		
	1–2 times per week	*n* = 413
	2–3 times per week	*n* = 537
	4–6 times per week	*n* = 360
	Daily	*n* = 77
**Hours of Training per Week**		
	</=5 h	*n* = 558
	5–10 h	*n* = 620
	10–15 h	*n* = 171
	>15 h	*n* = 35
**Training Plan**		
	Self-made	*n* = 477
	Created by a coach	*n* = 406
	Online plan	*n* = 42
	No Plan	*n* = 462
**Warm-Up**		
	1–5 min	*n* = 303
	5–10 min	*n* = 551
	>10 min	*n* = 371
	No warm-up	*n* = 162
**Activity Besides Fitness**		
**Physical Activity on Work**		
	Inactive (0–2 h)	*n* = 711
	Moderately active (2–4 h)	*n* = 298
	Active (4–8 h)	*n* = 287
	Highly active (>8 h)	*n* = 91
**Performing Other Sports**		
	Yes	*n* = 732
	No	*n* = 655
**Indicated Other Sport**		
	Running	*n* = 249
	Team Sports	*n* = 160
	Cycling	*n* = 155
	Swimming	*n* = 69
	Tennis	*n* = 52
	Mixed Martial Arts	*n* = 44
	Yoga	*n* = 32
	Climbing	*n* = 29
	CrossFit	*n* = 27
	Dancing	*n* = 26
	Horseback riding	*n* = 22

**Table 2:** Significant factors influencing the presence of pain in amateur fitness athletes.

Influencing Factor	*p*-Value
Gender	*p* < 0.001
Age	*p* = 0.1892
Obesity	*p* = 0.046
Years of training	*p* < 0.001
Training sessions per week	*p* < 0.001
Plan creation	*p* < 0.001
Other sports	*p* = 0.026

**Table 3:** First column: 49 exercises sorted by frequency of pain reported by the athlete and categorised as free-weight, bodyweight, machine, cardio and cable wire exercises (FW BB = free-weight with barbell; FW DB = free-weight with dumbbell; BW = bodyweight; M = machine; C = cardio; CA = cable wire). Second column: number of athletes indicated performing the exercise. The coloured fields indicate exercise frequency, the higher the frequency, the higher the colour saturation. Third column: *n* = having or having had pain related to exercise. This is the main focus of the table. The numbers are listed in descending order. Fourth column: *n* = performing the exercise but not having or having had any pain in relation to the exercise. Columns 5–7 indicate those who did not perform the exercises after a defined time or continued to perform them despite pain. Column 8 indicates the sum of all those athletes who no longer perform this exercise due to pain.

Exercise	N = Doing Exercise	Pain Indication: Yes	No	If, Yes, Still Continuing	Stop after 4 Weeks	Stop after 1 Year	Stop Less 1 Year	Sum of Demolition
Back squats (FW BB)	445 (f = 204; m = 241)	185	260	177	26	10	22	58
Deadlifts (FW BB)	461 (f = 207; m = 254)	147	314	106	13	12	13	38
Bench Press (FW BB)	432 (f = 160; m = 272)	136	295	92	18	3	20	41
Triceps dips (BW)	392 (f = 126; m = 265)	136	256	63	45	8	16	59
Strict Press (FW BB)	416 (f = 173; m = 242)	132	284	76	14	8	16	50
Treatmill (C)	503 (f = 241; m = 262)	130	372	72	23	13	21	57
Push up (BW)	478 (f = 217; m = 261)	118	359	82	18	6	8	32
Leg lifts (BW)	523 (f = 261; m = 262)	117	405	69	32	7	5	44
Lateral Rise (FW DB)	537 (f = 252; m = 285)	103	434	73	11	8	5	24
Leg press (M)	508 (f = 236; m = 272)	101	407	68	13	6	8	27
Biceps curls (FW DB)	495 (f = 198; m = 297)	101	394	57	19	10	8	37
Lunges (FW BB)	365 (f = 183; m = 182)	97	268	59	19	3	9	31
Triceps extension(FW DB)	481 (f = 228; m = 248)	85	401	43	21	10	2	33
Biceps curls (M)	454 (f = 166; m = 288)	80	373	47	20	6	3	29
Biceps curls (FW BB)	592 (f = 266; m = 325)	78	514	55	8	4	6	18
Crunches (BW)	594 (f = 282; m = 311)	72	522	48	8	5	3	16
Back Extension (BW)	478 (f = 222; m = 256)	72	406	48	13	5	5	23
Triceps Push up (BW)	434 (f = 196; m = 238)	71	362	45	12	4	8	24
Pull up (BW)	354 (f = 105; m = 213)	69	284	39	12	6	11	29
Strict press (M)	307 (f = 107; m = 200)	69	237	34	14	4	10	28
Dips on bench (BW)	301 (f = 131; m = 170)	64	237	29	24	3	4	31
Frontal lift (FW DB)	424 (f = 215; m = 209)	61	363	39	13	4	5	22
Leg curls (M)	464 (f = 201; m = 263)	60	403	34	11	7	7	25
Criss cross (BW)	491 (f = 256; m = 235)	58	432	44	7	3	2	12
Leg Extension (M)	464 (f = 202; m = 263)	57	406	29	16	3	5	24
Butterfly (M)	460 (f = 190; m = 270)	57	403	36	14	2	3	19
Front squats (FW BB)	263 (f = 95; m = 184)	55	207	35	7	3	5	15
Crosstrainer (C)	491 (f = 256; m = 235)	54	437	31	15	3	2	20
Pectoral press (M)	434 (f = 185; m = 249)	54	384	32	8	3	4	15
Butterfly (FW DB)	378 (f = 138; m = 200)	49	288	24	9	3	10	22
Row (C)	512 (f = 226; m = 286)	48	464	24	12	2	8	22
Stepper (C)	392 (f = 220; m = 173)	46	364	22	17	4	2	23
Row (FW BB)	420 (f = 183; m = 236)	44	376	8	7	2	4	13
Sit up inclined (BW)	352 (f = 144; m = 208)	44	308	17	16	3	3	22
Bike (C)	603 (f = 280; m = 323)	42	561	30	7	2	2	11
Lateral lift (M)	279 (f = 95; m = 184)	42	236	16	14	8	1	23
Pull ups (M)	355 (f = 142; m = 213)	40	315	30	6	6	4	16
Rowing cable (CA)	354 (f = 124; m = 230)	39	314	22	12	4	4	20
Triceps extension (CA)	481 (f = 184; m = 297)	37	443	25	6	3	2	11
Leg curls lying (M)	345 (f = 136; m = 209)	37	307	19	10	2	4	16
Triceps dips (M)	386 (f = 147; m = 239)	36	349	17	11	1	2	14
Russian twist (BW)	462 (f = 242; m = 220)	32	429	20	8	0	2	10
Abduction press (M)	424 (f = 232; m = 192)	32	392	22	3	2	1	6
Row (M)	462 (f = 193; m = 269)	30	432	21	5	0	2	7
Adduction press (M)	434 (f = 231; m = 204)	29	405	22	3	2	1	6
Single arm row (FW DB)	440 (f = 203; m = 237)	24	416	14	6	3	1	10
Calf press (M)	349 (f = 136; m = 213)	18	330	11	3	3	0	6
Triceps extension (M)	242 (f = 97; m = 145)	16	225	10	2	1	2	5
Single arm row (FW BB)	285 (f = 100; m = 185)	15	270	8	4	0	2	6

## Data Availability

The data presented in this study are available on request from the corresponding author. The data are not publicly available due to anonymized data records.

## Supplementary Materials

The following supporting information can be downloaded at: https://www.mdpi.com/article/10.3390/sports12010012/s1, Table S1: Questionnaire for personal and training-specific aspects.
