# Supplementary material for: Popular Gym Fitness Sport: An Analysis of 1387 Recreational Athletes Regarding Prone to Pain Exercises and the Corresponding Localisations
Source: Sports (Basel). 2023 Dec 29;12(1):12. doi: 10.3390/sports12010012 (PMC10821116; doi:10.3390/sports12010012)
Supplement: Supplementary file 1 [file sports-12-00012-s001.zip › sports-2766131-supplementary.pdf]

Supplementary file Tab. S 1: Questionnaire for personal and training-specific aspects (translated into English for publication)

Year of birth
Weight (in kg)
Height (in cm)
Gender
How long have you been working out at the gym, or how many years have you been working out at the gym in the past, if you are no longer working out at the gym?<br>O 1 – 2 years<br>O 2 – 3 years<br>O 3 – 7 years<br>O > 8 years
How often do you exercise per week (perhaps additionally at home)?<br>O 1 – 2 times a week<br>O 3 – 4 times a week<br>O 4 – 6 times a week<br>O daily
Do you follow a training plan (if so, who created the plan)?<br>O Yes, created on my own<br>O Yes, created online<br>O Yes, created by a coach<br>O No
Enter the total hours of your fitness training per week
Do you regularly participate in other sports besides fitness?
What other sports do you do besides fitness training and at what level (competitive, hobby)?
What is your level of physical activity at work?<br>O physically high active (> 8 hours)<br>O physically active (4 – 8 hours)<br>O physically moderate active (2 – 4 hours)<br>O physically inactive (0 – 2 hours)
Do you smoke?<br>O Yes<br>if yes how many cigarettes per year_____<br>if yes how many years ______<br>O No
Are you doing a warm – up regularly?<br>O Yes, 1 – 5 min<br>O Yes, 5 – 10 min<br>O > 10 min<br>O No
